# Comparison of the Visual Outcomes of Enhanced and Standard Monofocal Intraocular Lens Implantations in Eyes with Early Glaucoma

**DOI:** 10.3390/jcm12185830

**Published:** 2023-09-07

**Authors:** Jeoung Woo Nam, Jong Hoon Lee, Haowei Zhang, Mi Sun Sung, Sang Woo Park

**Affiliations:** Department of Ophthalmology, Chonnam National University Medical School and Hospital, Gwangju 61469, Republic of Korea

**Keywords:** enhanced monofocal intraocular lens, open-angle glaucoma, cataract surgery, visual outcomes

## Abstract

This study aimed to compare the efficacies and safety of enhanced and standard monofocal intraocular lenses (IOLs) in eyes with early glaucoma. Patients with concurrent cataracts and open-angle glaucoma (OAG) were enrolled. They underwent cataract surgery with IOL implantation. The comprehensive preoperative ophthalmic examination included the manifest refraction; monocular uncorrected distance visual acuity (UDVA), corrected distance visual acuity (CDVA), uncorrected intermediate visual acuity (UIVA), and uncorrected near visual acuity (UNVA); visual field (VF); and contrast sensitivity (CS); defocus curves and questionnaires were assessed three months postoperatively. Totals of 34 and 38 patients had enhanced and standard monofocal IOLs, respectively. The enhanced monofocal IOL provided better UIVA than the standard monofocal IOL (*p* = 0.003) but similar UDVA, CDVA, and UNVA. The enhanced monofocal IOL had more consistent defocus curves than the standard monofocal IOL, especially at −1 (*p* = 0.042) and −1.5 (*p* = 0.026) diopters. The enhanced monofocal IOL provided better satisfaction (*p* = 0.019) and lower spectacle dependence (*p* = 0.004) than the standard monofocal IOL for intermediate vision, with similar VF and CS outcomes. In conclusion, enhanced monofocal IOLs are recommended for patients with OAG because they provide better intermediate vision, higher satisfaction, and lower dependence on spectacles than standard monofocal IOLs, without worsening other visual outcomes.

## 1. Introduction

Glaucoma and cataracts are intrinsically age-related, and their comorbidity rate is high [1]. In a previous study, the most common ocular comorbidity in cataract patients was glaucoma [2]. As patients with glaucoma become older, the development of cataracts is inevitable and has also been associated with glaucoma medication [3,4]. Cataract surgery can significantly improve vision and quality of life for glaucoma patients, just as it does for nonglaucoma patients [5,6]. When choosing an intraocular lens (IOL) for glaucoma patients, it is essential to consider its potential impact on their visual acuity (VA), contrast sensitivity (CS), and visual field (VF) [7]. In the past, patients with glaucoma were not considered suitable candidates for multifocal IOLs because there was a fear that these IOLs would further reduce their CS and lead to other visual symptoms, such as photic phenomena [8,9,10]. Previous studies have shown that patients with glaucoma may experience decreased CS after bifocal IOL implantation [7,9,11]. However, in a prior study, a non-diffractive, wavefront-shaping extended depth-of-focus (EDOF) IOL provided good visual acuity and high patient satisfaction in eyes with mild, pre-perimetric open-angle glaucoma (OAG) [12]. In addition, EDOF IOLs have been reported to provide similar CS and VF outcomes to those of standard monofocal IOLs in cataract patients without ocular comorbidities [13,14,15].

The enhanced monofocal IOL is a new refractive IOL with an enhanced intermediate function. It uses the geometric, material, and corneal spherical aberration correction features of the standard monofocal IOL, as well as a higher-order aspheric profile on the anterior optic surface. The design of the enhanced monofocal IOL allows it to provide improved intermediate vision while maintaining the benefits of a standard monofocal IOL, such as clear distance vision. Other studies have shown no significant difference between the enhanced and standard monofocal IOLs in terms of CS, photic phenomena, and the incidence of side effects in eyes without ocular comorbidities [16,17]. Innovation in IOL technology has helped to overcome the limitations of earlier presbyopia-correcting IOLs [10,18,19]. Based on previous reports, we hypothesized that enhanced monofocal IOLs are viable for patients with concomitant cataracts and glaucoma. To the best of our knowledge, this study is the first in the literature to report the clinical results of enhanced monofocal IOLs in patients with glaucoma. This study aimed to compare the efficacies and safety of enhanced and standard monofocal IOLs in eyes with early OAG.

## 2. Materials and Methods

### 2.1. Study Design

This prospective, nonrandomized, observational, comparative study included patients with concurrent visually significant cataracts and OAG. As part of the preoperative counseling, all patients were educated on the inherent risks of surgery, the characteristics of IOLs, and some characteristics of their eyes. Patients chose IOLs based on their lifestyles, personalities, occupations, hobbies, and eye characteristics. The decision as to which IOL to choose is personal, and patients were given the information they needed to make an informed decision. All patients were informed of the study and provided informed consent for participation. This study adhered to the tenets of the Declaration of Helsinki and was approved by the Institutional Review Board of Chonnam National University Hospital (CNUH-2023-174). All study participants signed an informed consent form before enrollment.

### 2.2. Participants

This study included patients who were 50 years of age or older, had visually significant cataracts, and had medically controlled early OAG without central VF defects. The following inclusion criteria were used: (1) a visually significant cataract, defined as a nuclear cataract or cortical cataract grade ≥ 3 or posterior subcapsular cataract grade ≥ 2 on Lens Opacities Classification System III, which is the primary cause of vision impairment and self-reported vision worsening; (2) early OAG as defined by the presence of glaucomatous optic disc changes (vertical cup-to-disc ratio above 0.7, an inter-eye asymmetry of more than 0.2, or neuroretinal rim notching) with corresponding VF defects, open angle observed via gonioscopic examination, and reliable 30-2 VF test results (false-positive errors < 15%, false-negative errors < 15%, and fixation loss < 20%) with a mean deviation (MD) score of −6 dB or better; (3) medically controlled glaucoma, defined as no changes in the glaucoma medication regimen with intraocular pressure (IOP) at the target level (defined as IOP < 21 mmHg and at the individual IOP level at which the ophthalmologist believes that further optic nerve progression is unlikely to occur) and no structural or functional deterioration observed in the VF (determined via guided progression analysis II software of the Humphrey Field Analyzer, Carl Zeiss Meditec Inc., Jena, Germany) and optical coherence tomography (OCT) every 6 months within the last 1 year [20]; and (4) eyes without central VF defects, which are defined as the 4 most central points among the 76 testing points of the 30-2 VF test [21]. The exclusion criteria included amblyopia; an axial length above 26.0 mm; corneal astigmatism above 0.75 D; previous ocular surgery; previous ocular inflammation; acute ocular infection; diabetes mellitus with retinal changes; pseudoexfoliation syndrome; pathological miosis; possibility of floppy-iris syndrome; presence of corneal dystrophy; and any other ocular pathology that may limit visual function. Only one eye per patient was included in this study. If both eyes met the inclusion criteria, then only the first eye that underwent cataract surgery was enrolled in the study.

### 2.3. Preoperative Examination

Within 30 days of the surgery, all patients underwent a comprehensive preoperative ophthalmic examination, including their manifest refraction; monocular uncorrected distance visual acuity (UDVA at 4 m), corrected distance visual acuity (CDVA at 4 m), uncorrected intermediated visual acuity (UIVA at 66 cm), and uncorrected near visual acuity (UNVA at 33 cm) under photopic conditions (85 cd/m^2^) with the Early Treatment Diabetic Retinopathy Study charts; IOP measurement (Haag-Streit USA, Mason, OH, USA); topography (Pentacam; Oculus Optikgeräte GmbH Inc., Wetzlar, Germany); optical biometry (IOL master 700; Carl Zeiss Meditec, Dublin, CA, USA); global peripapillary retinal nerve fiber layer (pRNFL) thickness measurement via swept-source optical coherence tomography (OCT) (DRI OCT Triton; Topcon Corporation, Tokyo, Japan); VF test (Humphrey Field Analyzer; Carl Zeiss Meditec, Dublin, CA, USA); slit-lamp examination; gonioscopy; fundoscopy; and mesopic (3 cd/m^2^) pupillometry.

### 2.4. Surgical Technique

All the surgeries were performed by a single expert surgeon (PSW). Under topical anesthesia, a 2.8 mm corneal incision was made and a 5.0 mm sized capsulorrhexis was performed manually. Phacoemulsification was performed using the stop-and-chop technique with a phacoemulsification device (Centurion; Alcon Laboratories Inc., Fort Worth, TX, USA). All IOLs were implanted into bags. The incisions were sealed with hydrosutures. The postoperative target refraction was determined as the lowest myopic value after emmetropia using the Barrett Universal II formula. Each patient received topical antibiotic and anti-inflammatory prophylaxis and postoperative treatment with topical nonsteroidal anti-inflammatory, steroid, antibiotic, and artificial-tear eye drops.

### 2.5. Intraocular Lenses

The standard monofocal IOL (Tecnis model ZCB00; Johnson and Johnson Vision Care Inc., Santa Ana, CA, USA) is a single-piece, hydrophobic acrylic monofocal IOL with an aspheric anterior surface. The aspheric surface has a spherical aberration of −0.27 μm, which helps to reduce the spherical aberration to zero. The enhanced monofocal IOL (Tecnis Eyhance model ICB00; Johnson and Johnson Vision Care Inc.) is made of the same hydrophobic acrylic material and has the same overall geometry and dimensions as the standard monofocal IOL. Additionally, the enhanced monofocal IOL has a unique refractive optical design with a higher-order aspheric anterior surface, which produces a continuous increase in power. This design is intended to improve intermediate vision while maintaining distance vision comparable to that of a standard monofocal IOL. This is why it is called an enhanced monofocal IOL. It is based on refractive technology without diffractive rings and zones and is visually indistinguishable from the standard monofocal IOL.

### 2.6. Postoperative Examination

The UDVA, CDVA, UIVA, and UNVA under photopic conditions and postoperative manifest refraction were measured 3 months postoperatively. The corrected defocus curves were measured monocularly using defocusing lenses with a power range from 1.00 D to −4.00 D at 0.5 D intervals and a distance of 4 m. The lenses were inserted into a test frame to account for the manifest error in the refraction at distance [22]. The corrected CS was measured using the functional acuity contrast test of the Optec 6500 view-in test system (Stereo Optical Co., Inc., Chicago, IL, USA) with stimulus spatial frequencies between 1.5 and 18 cycles/degree under photopic (85 cd/m^2^) and mesopic (3 cd/m^2^) conditions with and without glare. Twelve white light-emitting diodes were used as the source of glare: 10.0 lx under photopic conditions and 1.0 lx under mesopic conditions. The VF test was performed using a Humphrey field analyzer with the Swedish Interactive Threshold Algorithm. The test was conducted under a 30-2 grid, with a white stimulus color, stimulus size III, and a background luminance of 31.5 apostilbs. Refractive corrections were performed for all eyes at a testing distance of 33 cm. Testing was repeated if the test results were unreliable. VF parameters, such as MD, pattern standard deviation (PSD), and visual field index (VFI), were assessed. The dB values of the four central test points of the VF 30-2 test program were anti-logged to obtain sensitivity on a linear scale. Subsequently, the mean of the four central test points was logged again to transform them back to a dB scale [23]. This value is defined as the central VF total deviation. Finally, patients completed questionnaires (File S1) to assess the experience of photic phenomena (glare, starbursts, and halos); their satisfaction with their overall, near, intermediate, and distance vision; and their dependence on spectacles for their near, intermediate, and distance vision. The questionnaires included illustrations to help patients identify photic phenomena. Patient satisfaction was rated on a scale from 1 to 5: 1, very dissatisfied; 2, dissatisfied; 3, neutral; 4, satisfied; and 5, very satisfied. Spectacle dependence was rated on a scale from 1 to 5: 1, never; 2, seldom; 3, about half the time; 4, usually; and 5, always [24,25].

### 2.7. Statistical Analysis

All statistical analyses were performed using SPSS version 26.0 (SPSS Inc., Chicago, IL, USA). Data are presented as means ± standard deviations. All VA measurements were converted to the logarithm of the minimum angle of resolution (logMAR) for data analysis. Student’s *t*-test, Pearson’s chi-square test, and Fisher’s exact test were used to verify the differences between the two groups. A paired *t*-test was used to compare the VF parameters before and after surgery in both groups. Statistical significance was set at *p* < 0.05.

## 3. Results

In this study, 72 eyes of 72 patients were evaluated. A total of 34 patients chose enhanced monofocal IOLs, and 38 patients chose standard monofocal IOLs. The demographic and preoperative clinical characteristics of all patients are shown in Table 1. There were no significant differences between the two groups based on age, sex, laterality, preoperative spherical equivalent (SE), axial length, mesopic pupil size, UDVA, CDVA, UIVA, UNVA, IOP, global pRNFL thickness, number of glaucoma medications, or VF parameters. In the enhanced monofocal IOL group and the standard monofocal group, 23.5% and 34.2% of patients, respectively, had UDVAs of 0.5 or better, but they reported visual impairment due to cataracts, so they underwent surgery. All surgeries proceeded smoothly, and all IOLs were successfully implanted in the capsular bag. There were no postoperative adverse events, such as cystoid macular edema, uncontrolled IOP, or endophthalmitis. All of the patients were able to complete the follow-up period. No patient had clinically significant posterior capsular opacification, and there was no significant difference in the corneal fluorescein staining score between the two groups at the 3-month follow-up.

Table 2 presents 3 months of postoperative visual outcomes for both groups. There were no significant differences in the UDVA, CDVA, or UNVA between the two groups. However, there were statistically significant differences in the UIVA; compared with the standard monofocal IOL group, the enhanced monofocal IOL group showed a significantly better UIVA (*p* = 0.003). The UIVA was 0.34 ± 0.10 logMAR for the enhanced monofocal IOL group and 0.41 ± 0.10 logMAR for the standard monofocal IOL group.

**Figure 1:**
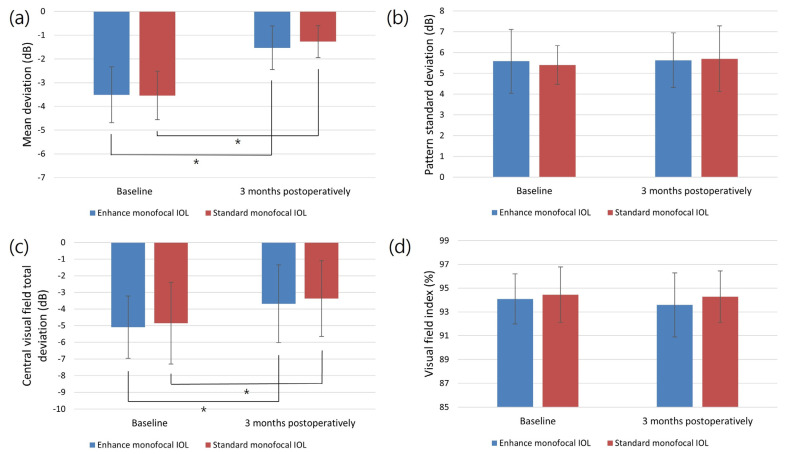
Visual field parameters of the two IOL groups at baseline and 3 months postoperation: (**a**) mean deviation; (**b**) pattern standard deviation; (**c**) central visual field total deviation; (**d**) visual field index (* = *p* < 0.05).

Figure 1 shows the changes in the VF parameters after surgery in both groups. The MD (*p* < 0.001 in both groups) and central VF total deviation (*p* = 0.017 for the enhanced monofocal IOL group and *p* = 0.010 for the standard monofocal IOL group) significantly improved at three months postoperation, compared with the baseline values for each group. The PSD and VFI showed no significant changes after surgery in either group. There were no statistically significant differences in any of the VF parameters between the enhanced and standard monofocal IOL groups at baseline or 3 months postoperation (*p* > 0.05).

**Figure 2:**
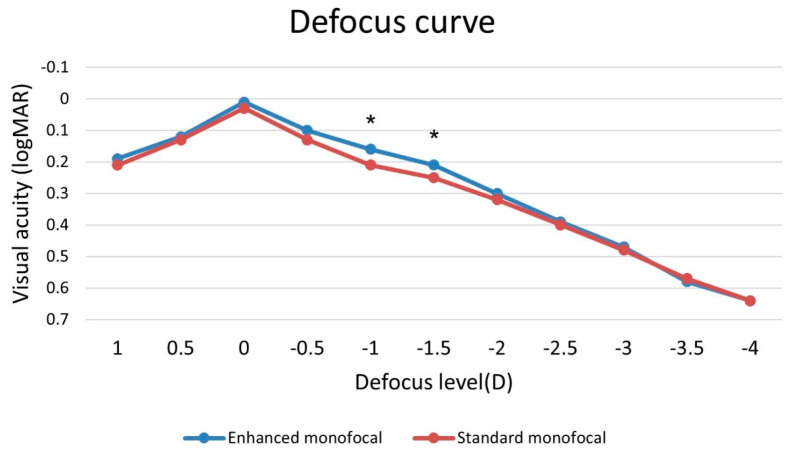
Corrected monocular defocus curve of the two IOL groups at 3 months postoperation (* = *p* < 0.05).

Figure 2 shows the corrected monocular defocus curves for the two groups at 3 months postoperation. Both curves peaked at a defocus of 0.00 D. VA decreased with increasing negative defocus. The enhanced monofocal IOL group had a smoother and wider defocus curve, especially within the intermediate defocus range (up to −1.50 D, corresponding to 66 cm). The enhanced monofocal IOL group showed significantly better defocus at −1.0 D (0.16 ± 0.08 logMAR for the enhanced monofocal IOL group vs. 0.21 ± 0.10 logMAR for the standard monofocal IOL group, *p* = 0.042) and −1.5 D (0.21 ± 0.10 logMAR in the enhanced monofocal IOL group vs. 0.25 ± 0.08 logMAR in the standard monofocal IOL group, *p* = 0.026) than the standard monofocal IOL group. This means that the enhanced monofocal IOL group had better intermediate vision than the standard monofocal IOL group.

**Figure 3:**
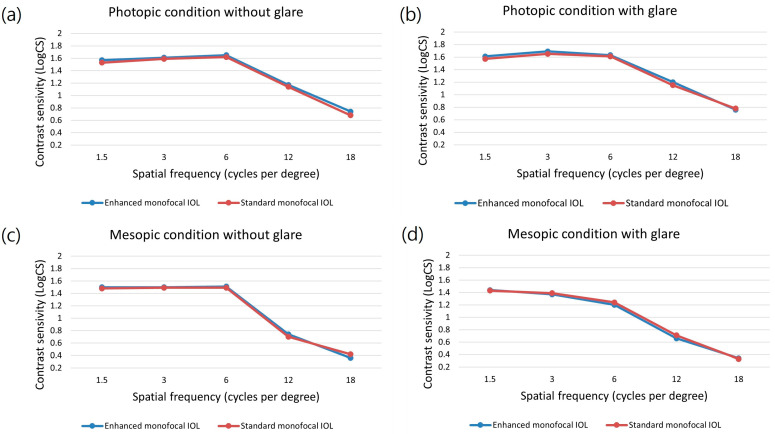
Corrected monocular contrast sensitivity of the two IOL groups at 3 months postoperation: (**a**) photopic condition without glare; (**b**) photopic condition with glare; (**c**) mesopic condition without glare; (**d**) mesopic condition with glare.

The monocular and corrected distance CS test results at 3 months postoperation under mesopic and photopic conditions with and without glare for eyes in the subgroup of patients are presented in Figure 3. The CS results showed no significant differences under any light condition between the enhanced and standard monofocal IOL groups for any spatial frequency (*p* > 0.05).

**Figure 4:**
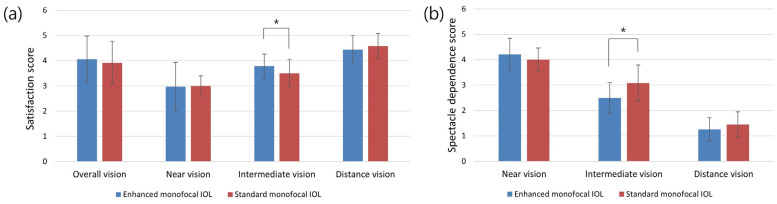
Questionnaire results for vision satisfaction and spectacle dependence of the two IOL groups at 3 months postoperation: (**a**) satisfaction score: 1, very dissatisfied; 2, dissatisfied; 3, neutral; 4, satisfied; and 5, very satisfied; (**b**) spectacle dependence score: 1, never; 2, seldom; 3, about half the time; 4, usually; and 5, always (* = *p* < 0.05).

Questionnaires on the perception of photic phenomena, visual satisfaction, and spectacle dependence were administered. No statistically significant differences were observed between the enhanced and standard monofocal IOL groups in terms of glare, starbursts, or halo perception (Table 3). In both groups, >90% of patients did not experience glare, halo, or starburst phenomena. Overall, visual satisfaction was observed in both groups. There was no statistically significant difference between the enhanced and standard monofocal IOL groups based on satisfaction or spectacle dependence for near or distance vision. However, for intermediate vision, the enhanced monofocal IOL group showed higher satisfaction (*p* = 0.019) and lower spectacle dependence (*p* = 0.004) than the standard monofocal IOL group (Figure 4).

## 4. Discussion

Cataract surgery is one of the most common surgical procedures globally. In recent years, it has evolved from simply removing clouded lenses to refractive surgery. Presbyopia-correcting IOLs such as accommodative, bifocal, trifocal, and EDOF IOLs have been introduced. These IOLs provide greater spectacle independence and an improved visual performance compared to standard monofocal IOLs [26]. However, some patients with multifocal IOLs may experience photic phenomena, such as glare and halo, or perceive reduced CS [27,28]. Patients with glaucoma have the same strong desires as their friends and family members who do not have glaucoma for a wide range of vision and reduced dependence on spectacles after cataract surgery. However, previous presbyopia-correcting IOL options, such as trifocal and bifocal IOLs, were considered unsuitable for patients with glaucoma because these IOLs can reduce CS and cause photic phenomena. Recently, the enhanced monofocal IOL has been introduced to provide excellent VA for distance vision while improving intermediate vision without the side effects that are typically associated with multifocal IOLs [29]. This study was designed to compare the efficacies and safety of enhanced and standard monofocal IOLs to provide improved vision for glaucoma patients.

The enhanced monofocal IOL has a unique refractive optical design with a higher-order aspheric anterior surface, which produces a continuous increase in power. The unique optical design of the enhanced monofocal IOL resulted in a more consistent defocus curve and a larger range of acceptable defocus values than those of the standard monofocal IOL. This is consistent with previous studies involving patients without ocular comorbidities [16,30]. As a result, the enhanced monofocal IOL can improve vision at intermediate distances, which is helpful for several modern daily life activities, such as using a computer screen, tablet display, or car dashboard, or playing sports [31]. This study shows that enhanced monofocal IOLs improve intermediate vision with increased depth of focus in glaucoma patients, as observed in patients without ocular comorbidities [16,32,33].

CS is closely associated with the ability to perform daily activities and provide valuable insights into quality-of-life assessments. Glaucoma first affects CS more than visual acuity [34]. CS, especially at mesopic levels, decreases early during the disease and is correlated with the severity of the VF loss in patients with glaucoma [35]. Therefore, additional CS loss is a main concern for patients with glaucoma. It is well known that CS decreases at higher spatial frequencies in eyes with multifocal IOLs [9,10]. Multifocal IOLs can reduce CS more than monofocal IOLs because one image is always blurred, and defocused light energy can create disturbances [36,37]. Therefore, multifocal IOLs are not recommended for glaucoma patients because they can further reduce the CS. To overcome these limitations, advancements have been made in IOL technology. A previous study found that eyes with EDOF IOLs have similar CSs to those with eyes with monofocal IOLs [10,13]. In this study, the monocular corrected distance CSs under mesopic and photopic conditions with or without glare were similar to those for the eyes with enhanced and standard monofocal IOLs, as reported in previous studies on patients without ocular comorbidities [16,30,32]. The enhanced monofocal IOL uses the same optical platform as the standard monofocal IOL, except for its unique anterior surface and a thickness difference of 1.5 microns with a diameter of nearly 2 mm in the optical center [38,39]. This subtle difference in optical design is not expected to have a significant impact on the CS in patients with glaucoma. The results of this study show that an enhanced monofocal IOL may be an appropriate choice for glaucoma patients who are concerned about reduced CS.

Patients with glaucoma who already have decreased VF sensitivity are at risk of further reductions due to cataract development. The deterioration in VF sensitivity by cataracts can be significantly improved via cataract removal and IOL implantation [40]. Several studies have reported improved MDs and unchanged VFIs after cataract surgery in glaucoma patients; however, the postoperative changes in the PSD, which vary from patient to patient, are still controversial [41,42]. In this study, the MD and central VF total deviation significantly improved after surgery using both IOLs. However, the PSD and VFI remained unchanged for both IOLs. As the type of IOL can affect the results of the VF test, it is important to carefully select the IOL for patients with glaucoma. Previous studies found that patients with multifocal IOLs had significantly lower MD values than those with monofocal IOLs [43,44]. However, a different study found that EDOF IOLs were comparable to monofocal IOLs based on both the MD and foveal threshold values [15]. In this study, there were no significant differences in the MD, PSD, or VFI between the two groups at 3 months postoperation. Additionally, there was no significant difference in the central VF total deviation value, which was strongly associated with the best-corrected VA and quality of life of the patients with enhanced and standard monofocal IOLs [45]. This suggests that enhanced monofocal IOLs may be appropriate for patients with glaucoma who are concerned about the deterioration of their VFs.

The enhanced monofocal IOL has an additional design in its central optics for intermediate vision. This design may increase the risk of positive dysphotopsia or photic phenomena, such as glare, halos, and starbursts. However, there was no significant difference in the patient-reported visual symptoms between the two IOL groups. This subtle difference in optical design is not expected to have a significant impact on photic phenomena. The majority of patients did not experience any significant problems or were not significantly bothered by photic phenomena. The enhanced monofocal IOL resulted in significantly better satisfaction with intermediate vision and lower spectacle dependence than the standard monofocal IOL. These results suggest that the enhanced monofocal IOL can provide satisfactory vision at both far and intermediate distances, with minimal photic phenomena.

To date, patients and surgeons have been reluctant to use multifocal IOLs not only because of concerns about their disadvantages, but also because of the potential risk of glaucoma progression. Therefore, it is important to choose an IOL that is less affected by glaucoma progression. Especially in younger patients with a longer duration of disease progression, increased emphasis on presbyopia correction is associated with a greater risk of visual impairment in the future if glaucoma progresses. It would be ideal if an IOL could perfectly correct presbyopia and its efficacy was not affected by the glaucoma severity. However, such an IOL has not yet been developed. It is necessary to make a balanced choice between the correction of presbyopia and the risk of glaucoma progression. In this regard, the enhanced monofocal IOL may be an appropriate and balanced option for glaucoma patients who will undergo cataract surgery. The enhanced monofocal IOLs share more similar optical features to standard monofocal IOLs than other presbyopia-correcting IOLs. Although the presbyopia correction is less than that of other presbyopia-correcting IOLs, it provides improved intermediate vision that is satisfactory to patients, and it minimizes the disadvantages of presbyopia-correcting IOLs. Our study did not find any other negative effects of the enhanced monofocal IOL in glaucoma patients, but there is a potential risk. The risks and benefits of using enhanced monofocal IOLs in glaucoma patients should be carefully considered.

The present study had several limitations. The main limitation of the study was the lack of randomization, as patients were divided according to their personal preferences after comprehensively considering their own daily lives. However, specific information on the patients’ primary daily distances or occupations was not collected. Patients already knew the properties of the IOL, which was intended to enhance intermediate vision. This may have affected the patients’ responses to the questionnaire. Additional studies with randomization and specific information on the patients’ lives would be helpful for evaluating the effectiveness of the enhanced monofocal IOL in a more practical setting. Second, even though the central VF was evaluated using the central portion of the 30-2 visual field test, a more precise evaluation, such as the 10-2 VF test, is needed for a more appropriate central VF function. Third, although there was no significant difference in the mesopic pupil size between the two groups preoperatively, it is considered necessary to evaluate the pupil size postoperatively, as the pupil size can affect the optical characteristics of the IOL. Fourth, although an uncorrected defocus curve may be representative of the real-life visual function as perceived by patients, a corrected defocus curve was obtained to better represent the inherent characteristics of IOLs. Additional studies that use uncorrected defocus curves may yield more practical outcomes. Fifth, a 3-month follow-up period is not long enough to assess whether medically controlled OAG will progress or whether PCO will develop. Analysis after long-term follow-up may yield different results. Sixth, this study was conducted monocularly, but the satisfaction questionnaire was conducted in daily life, so the satisfaction may vary depending on the condition of the opposite eye. Therefore, further studies are needed to assess the impact of bilateral IOL insertion on patient satisfaction. Finally, this study involved patients with early OAG, preserved central VFs, and a low risk of glaucoma progression. Therefore, it may be difficult to apply our results to all glaucoma patients. Based on the current results, further studies may be needed to assess the long-term visual outcomes of enhanced monofocal IOLs in patients with varied glaucoma severities.

In conclusion, we suggest enhanced monofocal IOLs for patients with OAG because they provide better intermediate vision, higher satisfaction, and lower dependence on spectacles than standard monofocal IOLs, without worsening other visual outcomes.

## Figures and Tables

**Table 1:** Demographic and preoperative characteristics of the two IOL groups.

Variable	Enhanced Monofocal IOL (n = 34)	Standard Monofocal IOL (n = 38)	*p*-Value
Age (years)	68.76 ± 6.96	66.00 ± 7.59	0.113
Sex (male/female)	18/16	18/20	0.979
Laterality (right/left)	19/15	18/20	0.471
SE (diopter)	0.46 ± 1.67	0.75 ± 1.80	0.488
Axial length (mm)	24.74 ± 0.77	24.57 ± 0.84	0.370
Mesopic pupil size (mm)	3.76 ± 0.82	3.90 ± 0.83	0.478
UDVA (logMAR)	0.39 ± 0.17	0.36 ± 0.20	0.612
CDVA (logMAR)	0.22 ± 0.16	0.23 ± 0.14	0.698
UIVA (logMAR)	0.42 ± 0.14	0.43 ± 0.17	0.880
UNVA (logMAR)	0.56 ± 0.14	0.52 ± 0.14	0.183
IOP (mmHg)	12.76 ± 1.94	12.97 ± 1.81	0.638
Global pRNFL thickness (μm)	86.29 ± 10.67	84.53 ± 10.44	0.480
Number of glaucoma medications	1.47 ± 0.51	1.45 ± 0.50	0.846
30-2 VF parameters			
MD (dB)	−3.51 ± 1.18	−3.54 ± 1.02	0.903
PSD (dB)	5.58 ± 1.53	5.40 ± 0.93	0.562
VFI (%)	94.09 ± 2.11	94.45 ± 2.34	0.499
Central VF total deviation (dB)	−5.09 ± 1.87	−4.85 ± 2.45	0.645

SE: spherical equivalent; UDVA: uncorrected distance visual acuity; CDVA: corrected distance visual acuity; UIVA: uncorrected intermediate visual acuity; UNVA: uncorrected near visual acuity; logMAR: logarithm of the minimal angle of resolution; IOP: intraocular pressure; pRNFL: peripapillary retinal nerve fiber layer; VF: visual field; MD: mean deviation; PSD: pattern standard deviation; VFI: visual field index.

**Table 2:** Visual acuity outcomes of the two IOL groups at 3 months postoperation.

Variable	Enhanced Monofocal IOL (n = 34)	Standard Monofocal IOL (n = 38)	*p*-Value
UDVA (logMAR)	0.05 ± 0.07	0.05 ± 0.06	0.983
CDVA (logMAR)	0.01 ± 0.04	0.02 ± 0.04	0.678
UIVA (logMAR)	0.34 ± 0.10	0.41 ± 0.10	**0.003**
UNVA (logMAR)	0.50 ± 0.10	0.53 ± 0.08	0.168
SE (diopter)	−0.25 ± 0.22	−0.21 ± 0.36	0.605

UDVA: uncorrected distance visual acuity; CDVA: corrected distance visual acuity; UIVA: uncorrected intermediate visual acuity; UNVA: uncorrected near visual acuity; logMAR: logarithm of the minimal angle of resolution; SE: spherical equivalent. Factors with statistical significance are shown in bold.

**Table 3:** Questionnaire results for photic phenomena of the two IOL groups at 3 months postoperation.

Variable	Enhanced Monofocal IOL (n = 34)	Standard Monofocal IOL (n = 38)	*p*-Value
Glare (yes/no)	2/32	3/35	>0.999
Starbursts (yes/no)	1/33	2/36	>0.999
Halos (yes/no)	2/32	1/37	0.599

## Data Availability

The datasets used and/or analyzed during the current study are available from the corresponding author upon reasonable request.

## Supplementary Materials

The following supporting information can be downloaded at https://www.mdpi.com/article/10.3390/jcm12185830/s1, File S1: Cataract surgery satisfaction questionnaire.
