# Supplementary material for: Comparison of the Visual Outcomes of Enhanced and Standard Monofocal Intraocular Lens Implantations in Eyes with Early Glaucoma
Source: J Clin Med. 2023 Sep 7;12(18):5830. doi: 10.3390/jcm12185830 (PMC10531790; doi:10.3390/jcm12185830)
Supplement: Supplementary file 1 [file jcm-12-05830-s001.zip › jcm-2549848-supplementary.pdf]

< Cataract Surgery Satisfaction Questionnaire >

Please check the box that best reflects the content of each question.

**Do you experience glare?**	○ Yes	○ No
**Do you experience starbursts?**	○ Yes	○ No
**Do you experience halos?**	○ Yes	○ No

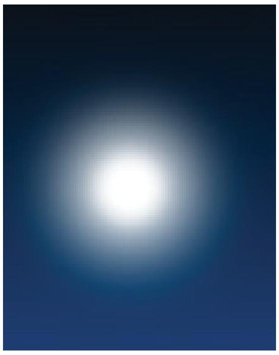

Glare

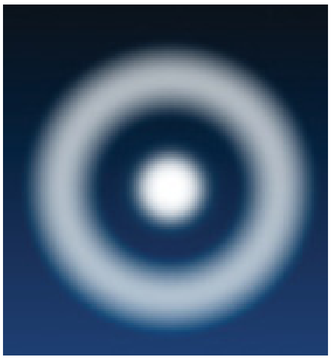

Halo

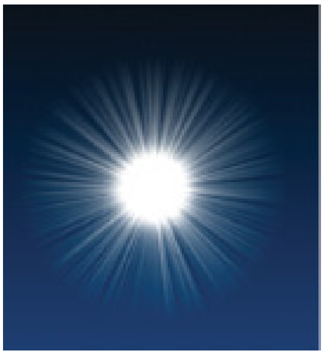

Starburst

	Never	Seldom	About half the time	Usually	Always
Do you need glasses for near vision (book, smart phone)?	○ 1	○ 2	○ 3	○ 4	○ 5
Do you need glasses for intermediate vision (computer monitor)?	○ 1	○ 2	○ 3	○ 4	○ 5
Do you need glasses for distance vision (TV)?	○ 1	○ 2	○ 3	○ 4	○ 5

Please check the box that best reflects the content of each question.

	Very dissatisfied	Dissatisfied	Neutral	Satisfied	Very satisfied
**How satisfied are you with your overall visual acuity?**	○ 1	○ 2	○ 3	○ 4	○ 5
**How satisfied are you with your near visual acuity (smart phone)?**	○ 1	○ 2	○ 3	○ 4	○ 5
**How satisfied are you with your intermediate visual acuity (computer monitor)?**	○ 1	○ 2	○ 3	○ 4	○ 5
**How satisfied are you with your distance visual acuity?**	○ 1	○ 2	○ 3	○ 4	○ 5
